# Legg-Calvé-Perthes disease– surgical treatment options

**DOI:** 10.1007/s00402-025-05801-3

**Published:** 2025-03-12

**Authors:** Sebastian Braun, Stefanie Adolf, Marco Brenneis, Friedrich Boettner, Andrea Meurer

**Affiliations:** 1 Charité - University Medicine Berlin, Berlin, Germany; 2 St. Josefs Hospital, Wiesbaden, Germany; 3 Hospital for Special Surgery, New York, USA; 4 Medical Park Klinik, Bad Wiessee, Germany

**Keywords:** Legg-Calvé-Perthes disease, Epidemiology, Surgical treatment, Containment-restoring procedures, Femoral varus osteotomy, Salter's innominate osteotomy, Triple pelvic osteotomy, Non-containment-restoring procedures

## Abstract

Legg-Calvé-Perthes disease (LCPD), is a rare avascular osteonecrosis of the proximal femur usually occurring in children between 5 and 10 years of age. The cause of ischemia leading to necrosis of the femoral head remains unknown. The goal of surgical treatment for LCPD is to improve the containment of the femoral head to restore the function of the hip joint and prevent further damage to the femoral head leading to premature hip osteoarthritis. Although a causal therapy is not available, the main aim is to maintain or restore the containment of the affected hip joint. The specific surgical treatment depends on the patient’s age at onset, the stage, and severity of the disease. In early stages of the disease, the most common surgical option is a containment-restoring procedure such as femoral varus osteotomy (FVO), Salter’s innominate osteotomy (SIO), and triple pelvic osteotomy (TPO). Moderate forms of LCPD show good results after treatment with either FVO or SIO, severe cases are recommended to be treated with FVO combined with either SIO or TPO to provide good outcomes. In later stages with increased damage to the femoral head, surgical options may include non-containment-restoring procedures to help symptom relief or restore anatomical and biomechanical features to a certain extend e.g., femoral valgus extension osteotomy or trochanter apophyseodesis. Due to the complexity of surgical interventions and the challenging nature of LCPD it is essential to consult with an experienced surgeon in pediatric orthopedics to determine the best treatment course for the patient.

## Introduction

More than a century after its initial description in 1910, Legg-Calvé -Perthes disease (LCPD) continues to raise many questions. Following hip dysplasia, LCPD is the most common disease of the hip joint in childhood [1]. It is an avascular osteonecrosis of the proximal femoral epiphysis and a subsequent ossification disorder in children with skeletal immaturity [2]. LCPD is the most common osteonecrosis in children. The cause is still unknown, but various pathogenetic factors are discussed [3]. Clinical signs of the disease are hip as well as knee pain, reluctance to move, and limited range of motion, especially in abduction and internal rotation of the hip joint [4]. The disease is self-limiting and progresses in defined stages over a period 2–5 years. Although a causal therapy is not available, the aim of current therapeutic approaches is to increase (or maintain a good) range of motion, to preserve the femoral head, to limit mechanical stress on the hip joint and eventually to restore the containment surgically in order to reduce the risk of early hip osteoarthritis (OA) [5]. During progression of LCPD, patience and discipline regarding the individual therapy is required from the young patients, their families and therapists. In this review we will present a brief overview of important characteristics of the disease, followed by a description of the different established surgical treatment options. Today’s surgical procedures can be divided into containment-restoring and non-containment restoring procedures. The containment-restoring procedures include femoral varus osteotomy (FVO), Salter’s innominate osteotomy (SIO), triple pelvic osteotomy (TPO), periacetabular osteotomy (PAO) and head reduction osteotomies (FHRO). In more severe cases, where containment restoring options are no longer recommended, surgeons might have to resort to non-containment-restoring procedures for symptom improvement, such as Morscher’s femoral neck lengthening osteotomy, trochanter apophyseodesis or total hip arthroplasty (THA) for skeletally mature deformities.

## Methodology

For this comprehensive narrative review, an extensive analysis of literature concerning the surgical treatment LCPD was undertaken. Our search strategy involved querying the PubMed bibliographic database with relevant keywords such as ‘Legg-Calvé-Perthes Disease’, ‘surgical treatment’, and ‘outcome analysis’. We conducted both unrestricted free-text and specific term searches, integrating them with Boolean operators to ensure a broad yet relevant scope of literature. Special emphasis was placed on the most recent publications to capture the latest advances and opinions in the field. This review also included a detailed examination of reference lists from key articles to ensure a thorough coverage of seminal works and prevailing theories. Our methodology did not involve a systematic meta-analysis of data, reflecting our intention to encapsulate a wide spectrum of clinical studies and expert opinions, thus providing a comprehensive narrative synthesis.

The authors of this review are seasoned clinicians with distinct and extensive backgrounds in the surgical treatment of LCPD. Their cumulative experiences span several decades and are deeply rooted in both academic and clinical settings. This depth of clinical involvement ensures a robust and nuanced understanding of the disease, significantly enriching the perspectives and recommendations put forth in this review. Their hands-on experience in managing complex cases provides a practical grounding to the theoretical insights discussed, thereby enhancing the authenticity and applicability of the review’s content.

### Epidemiology

In the Caucasian population, the incidence of LCPD is 5–10:100,000 new cases per year [6]. The study by Purry et al. [7] showed that LCPD shows different ethnical expression patterns and occurs mainly in the Caucasian population. In comparison the incidence in the African community is 0.45:100,000. Barker et al. [8] found evidence that lower social classes were associated with an increasing incidence of the disease of 15.6:100,000 in the Liverpool area. LCPD also shows gender-specific differences: boys seem to be affected by LCPD 3 to 5 times more often than girls (♂:♀, 3–5:1). Bilateral involvement of the hip joints is seen in 10–24% of cases, with no difference in incidence between the right and left hip [9]. Usual onset of LCPD is between the age of 3 to 12 with a peak between the 5 and 7 years. An occurrence in children under the age of 2 years and above the age of 10 years is rare and must be considered critically for differential diagnosis [1].

### Pathophysiology and morphology

More than 100 years after its discovery, the etiology of the proximal femoral epiphysis ischemia occurring in LCPD remains unknown, though various theories exist (Table 1). LCPD is characterized by its progression through different stages: initial, condensation, fragmentation, reconstitution, and healed stage.

**Table 1:** Etiology– possible theories

Theory	Features
Genetic	• Mutation in type-II collagen gen (COLA2A1) [10] • Polymorphism in endothelial nitric oxide synthase (eNOS) gene [11] • Family cluster (35-fold increased risk) [12]
Vascular	• Intraarticular or intraosseous pressure increase [13, 14] • Obliteration or atrophy of blood vessels supplying the femoral head [15, 16]
Coagulation disorder	• Thrombophlebias (factors C or S deficiency), factor V Leiden mutation, elevation of lipoproteins in serum, G20210A prothrombin mutation, factor VIII elevation [17]
Growth factors	• Changes in IGF-1 (insulin-like growth factor 1) [18]
Environmental factors	• Socio-economic deprivation, passive smoking, malnutrition [19, 20] • Retarded skeletal age (small for gestational age– SGA) [21, 22]

### Classifications

With the discovery of x-rays in 1895, radiological imaging as an objective diagnostic method of LCPD became available. Since then, many classifications have been developed, which have different prognostic and diagnostic value. The most common classifications are shown in Tables 2 and 3. The different classification systems are important for assessing the severity and thus for determining the prognosis of LCPD. The first published classification of LCPD by Waldenström [23] in 1920 solely deals with morphological changes on plain x-rays of the disease’s natural course without a prognostic estimation. In 1971, Catterall [24] published a four-staged classification system describing the extent of necrosis of the femoral epiphysis based on the quadrants involved. This classification was supplemented with the “head at risk signs” to provide an estimation of the course of the disease by prognostically unfavorable signs [24]. The Catterall classification is well known, but its clinical value is rather low. An additional classification published by Salter and Thompson [25] (1984) refers to the extent of the subchondral osteolytic zone which is described as an indicator for the final femoral head defect. In contrast to the Catterall classification, Salter and Thompson believed that by including the subchondral osteolytic zone, grading at earlier stages would be possible. Without the presence of subchondral fracture, there is no resorption, and healing subsequently goes without defect. The problem with this classification is that subchondral fracture can only be found during the first four weeks of the disease. Subsequently, the prognostic value of this classification is limited. In 1992, Herring et al. [26] published another classification to assess the long-term prognosis of LCPD. This classification is the most widely used, today. It predicts final outcome at onset of fragmentation stage and has a good prognostic value. Herring divided the femoral head into three pillars and the appearance of the lateral pillar is assessed with respect to its height at onset of fragmentation. An intact lateral column is prognostically favorable, since no significant deformations of the femoral head and no subluxation phenomena are expected to develop. If the lateral pillar is affected, a reduction of the lateral support, an increased lateralization and subluxation, and an increased loading of the epiphysis leading to deformity can be assumed. Most recently, Herring added the sub-type B/C (“boarder group”) to his classification [27]. The Elizabethtown classification provides a detailed framework that categorizes the stages of Perthes disease based on radiographic findings, facilitating a more precise assessment and tailored management strategies for each phase of the disease’s progression [28]. This dynamic classification aids in fine-tuning treatment approaches tailored to individual patient profiles, thus optimizing therapeutic outcomes. Today, Stulberg classification [29] (1981) is considered to be the reference standard for estimating long-term outcomes of LCPD. He divided the final stage of the disease into five groups according to hip joint morphology and congruence. The classification system describes the extent of residual deformity of the femoral head and acetabulum with increasing severity within the five groups. Simultaneously, the risk of developing OA at skeletal maturity increases. The sphericity of the femoral head and the congruence of head and acetabulum determine the long-term prognosis (30–60 years). In class I and II the risk for development of hip OA is low, in class III and IV there is a significant increased risk for development of mild to moderate hip OA in late adulthood, and in class V there is a high risk for development of severe hip OA before the age of 50 years [29].

**Table 2:** Classification of Legg-Calvé-Perthes disease

Classification	Features
**Extent of necrosis, Catterall (24)**	
Grade I	Affection of one quarter (< 25%) of the femoral head - anterolateral quadrant
Grade II	Affection of anterior third to half (< 50%) of the head
Grade III	Affection of three quarters of the head (< 75%), only dorsal portion intact
Grade IV	Affection of entire femoral head
**Head at risk signs**,** Catterall (24)**	
Lateral calcification	Calcification lateral to femoral head
Subluxation	Lateralization of femoral head
Metaphyseal affection	Osteonecrosis of adjacent metaphysis
Horizontalization of the physis	Horizontal alignment of the physis
Gage sign	Triangular osteoporosis at the lateral femoral head
**Subchondral fracture**,** Salter and Thompson (25)**	
Group A	Subchondral fracture involves < 50% of the femoral head dome (corresponds to Catterall groups I and II)
Group B	Subchondral fracture involves > 50% of the femoral head dome (corresponds to Catterall groups III and IV)
**Lateral pillar Classification**,** Herring (26**,** 27)**	
Group A	Lateral pillar intact
Group B	> 50% of the lateral pillar intact
Group B/C	50% of lateral pillar intact with poor ossification
Group C	< 50% of the lateral pillar intact
**Residual deformity**,** Stulberg (29)**	
Class I	Spherical congruency: round femoral head ◊ no coxa magna, normal acetabulum and neck
Class II	Spherical congruency: round femoral head ◊ coxa magna or steep acetabulum or short neck
Class III	Aspherical congruency: ovoid femoral head
Class IV	Aspherical congruency: flat femoral head ◊ coxa magna or steep acetabulum
Class V	Aspherical incongruency: flat femoral head ◊ normal acetabulum and no coxa magna

**Table 3:** Elizabethtown classification of Legg-Calve-Perthes disease [28]

Stage	Radiological Findings	Therapeutic indications	Clinical importance
Ia	Sclerosis without loss of height; intact epiphysis.	Activity modification, physical therapy.	Early stage; maintain joint mobility.
Ib	Sclerosis with minor height loss; no fragmentation.	Continue conservative management and monitoring.	Progressing disease; may need more aggressive intervention soon.
IIa	Initial fragmentation with few fissures.	Begin containment strategies to prevent deformity.	Critical for positive influence on disease trajectory.
IIb	Advanced fragmentation; no new bone.	Aggressive containment, possibly surgical.	High risk of deformation; intervention critical to prevent disability.
IIIa	Early porous new bone covering less than one-third of epiphysis.	Maintain or initiate containment; supportive care.	Recovery phase begins; focus on regeneration support.
IIIb	Normal textured new bone covering more than one-third of epiphysis.	Reduce intervention intensity; continue rehabilitation.	Recovery progressing; optimize function and minimize deformities.
IV	Complete revascularization; potentially residual deformities.	Physical rehabilitation; correct deformities if needed.	Monitor for osteoarthritis; maximize function and quality of life.

MRI plays a role in the early phase of the disease when native radiological signs are not yet present. Diffusion-weighted imaging (DWI) and perfusion MRI have shown significant prognostic value in predicting disease severity and mid-term radiographic outcomes of early LCPD and consequently guiding treatment decisions [30]. An increased apparent diffusion coefficient (ADC) in the femoral metaphysis is associated with a worse prognosis, correlating with higher Stulberg classifications at skeletal maturity [30]. Furthermore, it has been shown that a high ADC of the metaphysis on DWI without gadolinium enhancement or reduced epiphyseal perfusion in the early fragmentation stage on (gadolinium enhanced) perfusion MRI sequences are associated with an unfavorable prognosis [31–33]. Similarly, perfusion MRI can identify areas of reduced epiphyseal vascularity, which are linked to early fragmentation and femoral head deformity [34]. These advanced imaging techniques can detect early ischemic changes before they appear on radiographs, enabling earlier intervention and treatment planning. Despite their utility, MRI findings have yet to be directly correlated with specific surgical outcomes, limiting their role in optimizing treatment strategies. Currently, patients diagnosed with LCPD in the early stages via MRI typically undergo nonoperative management, as many cases do not meet radiographic criteria for surgical interventions. However, MRI may help stratify high-risk patients who could benefit from early containment surgery before radiographic fragmentation occurs [35, 36]. Future research should focus on correlating MRI parameters with surgical success rates to refine treatment algorithms and improve long-term joint preservation.

### Surgical indication and prognosis

The prognostic value of the individual clinical and radiological parameters is crucial for the choice of therapy (Table 4). The greatest prognostic value is given by age at onset, the condition of the lateral pillar, the extent of necrosis, subluxation, lateral calcification, mobility, and gender. According to a prospective multicenter study by Herring [37], surgical intervention (FVO or SIO) significantly improves outcomes in children over 8 years of age with Herring B or B/C hips, compared to nonoperative management. In contrast, younger children (≤ 8 years old) with Herring B hips tend to have favorable outcomes regardless of treatment, suggesting that surgical intervention in this group may not be necessary. Additionally, Herring C hips, regardless of age, demonstrate poor long-term outcomes, with no clear advantage of surgery over conservative treatment [37]. These findings emphasize that in older children (> 8 years) with Herring B or B/C hips, early surgical containment may help preserve joint congruency and improve long-term femoral head sphericity, whereas treatment decisions in younger children should be more individualized. Similarly, the extent of necrosis according to Catterall is important because small areas of necrosis (Catterall I and II) are associated with better outcomes than more extensive findings, such as Catterall III and IV [27, 38]. However, the “head at risk signs,” particularly lateral calcification and subluxation, have a greater negative prognostic value. These two factors are signs of containment loss [3, 24]. Furthermore, hip mobility and gender have an important influence on prognosis. Better hip range of motion is associated with a more favorable prognosis for reconstitution. Although LCPD is more common in boys, studies have shown that girls tend to have worse outcomes, which may be attributed to differences in skeletal maturation and remodeling potential [26, 29]. Girls reach skeletal maturity earlier than boys, resulting in a shorter period for femoral head remodeling, which may contribute to higher rates of residual deformity and poorer functional outcomes [39]. While treatment strategies remain largely similar between sexes, girls with LCPD may require closer monitoring for early joint deterioration, and in some cases, earlier surgical intervention to maximize containment before growth plate closure. The duration of the natural course is dependent on the extent of necrosis and thus indirectly on the age at onset because reparative and remodeling abilities are largely determined by the age at initial diagnosis. With increasing age, the diameter of the femoral head and thus the volume of necrosis increases during the course of the disease [40]. Average duration of the disease varies between 2 and 5 years [3]. A systematic review of LCPD surgical procedures by Caldaci et al. [41] concluded that surgical treatment in patients older than 6 years with Herring B and B/C hips resulted in a high percentage (Herring B: 70%, and Herring B/C: 57%) of favorable Stulberg I and II type hips, whereas in Herring C hips only 38% resulted in Stulberg I and II types, although with a slight advantage for patients between 6 and 8 years of age. The study findings confirmed that early surgical treatment during the fragmentation stage or earlier, which is possible in patients with a younger age at onset of LCPD, is the most important indicator for a positive surgical outcome. Additionally, the degree of initial disease severity and an appropriate preoperative hip range of motion were also identified as crucial factors for success [41]. However, current literature lacks a standardized, evidence-based algorithm to guide procedure selection based on disease severity, age at onset, and hip morphology. To address these gaps, we have integrated a structured decision-making framework, outlining optimal surgical approaches based on patient age, disease classification, and expected outcomes, ensuring this review serves as a practical resource for pediatric orthopedic surgeons.

**Table 4:** Prognostic factors [3, 42, 43]

Prognostic factor	Prognostic value	favorable	unfavorable
Age at onset (skeletal age)	+++	< 8 years	> 8 years
Lateral calcification	++	none	present
Subluxation	++	none	present
Herring Classification	++	A, B	B/C, C
Range of motion	++	good	poor
Gender	++	male	female
Metaphyseal affection	+	none	present

Maintenance and/or restoration of a congruent joint with good containment is the most important therapeutic goal to avoid secondary damage from eccentric joint loading. Table 5 shows the therapeutic options for the treatment of LCPD.

**Table 5:** Therapy options for Legg-Calve-Perthes disease [42]

Age at onset (skeletal age)	Features	Therapy
< 8 years	Good hip mobility, Containment preserved No risk signs	Physical therapy Traction therapy
< 8 years	Good hip mobility, Containment preserved Initial risk signs	Physical therapy Traction therapy Loss of containment: femoral varus osteotomy
< 8 years	Limited hip mobility, Loss of containment	femoral varus osteotomy combined with Salter’s innominate osteotomy (if necessary)
> 8 years	Limited hip mobility, Loss of containment	Triple pelvic osteotomy combined with femoral varus osteotomy (if necessary)
All ages	Hinge abduction phenomenon, abduction < 30°	Femoral valgus osteotomy

## Therapy

### Conservative therapy

In the initial phase of LCPD, when hip mobility is preserved and radiographic imaging confirms joint congruence with the epiphysis centered within the acetabular cup, conservative therapy remains the first-line treatment approach. Conservative management aims to maintain joint mobility, reduce mechanical stress, and optimize conditions for natural femoral head remodeling. Core components of conservative treatment include structured physiotherapy with gentle range-of-motion exercises, traction therapy where indicated, and activity modifications such as limiting high-impact activities. Analgesia may be administered as needed, and moderate unloading of the affected limb can be considered, though its efficacy remains controversial, particularly in active children [3, 42]. Recent studies and clinical consensus have further refined non-surgical treatment strategies, emphasizing self-management approaches that include patient and family education, physiotherapy-led exercise regimens, and close clinical monitoring [44]. The 2024 clinical consensus recommendations by Galloway et al. [45] highlight the importance of structured rehabilitation programs, supervised weight-bearing modifications, and tailored exercise protocols depending on disease stage. Recommendations strongly support the use of hydrotherapy, controlled cardiovascular exercise, and stretching programs to preserve joint function while minimizing joint stress. Notably, while bracing and orthoses were historically used, current evidence suggests they offer no additional benefit over active physiotherapy and are no longer recommended [37, 46]. Additionally, adjunctive interventions such as targeted Botox injections for iliopsoas and adductor muscle release may aid in improving hip range of motion in select cases [47]. Multidisciplinary care, involving orthopedic specialists, physiotherapists, and rehabilitation teams, plays a crucial role in guiding conservative treatment and identifying cases where surgical intervention may be warranted. While most LCPD cases are initially managed non-operatively, surgical intervention is considered when there is progressive femoral head deformity, persistent range-of-motion limitations, or loss of containment despite conservative measures.

Socioeconomic status has been shown to influence both the incidence and outcomes of LCPD, with lower status being associated with delayed diagnosis, limited access to specialized care, and worse long-term joint function [20]. Patients from lower socioeconomic backgrounds may face barriers to timely intervention, rehabilitation, and post-operative follow-up, which can negatively impact outcomes. To address these disparities, treatment strategies should include enhanced early screening programs in underserved populations, improved access to physiotherapy and rehabilitation services, and multidisciplinary care coordination [44]. Additionally, patient education and financial support programs may help mitigate the challenges associated with long-term disease management, ensuring more equitable outcomes regardless of socioeconomic background.

### Surgical treatment options

**Fig. 1:**
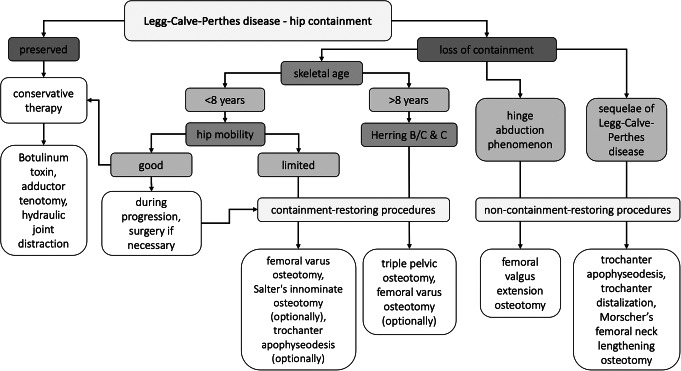
Therapy algorithm for Legg-Calvé-Perthes disease [41]

Surgical therapy follows the principle of improving or restoring containment and thus centering the hip joint. Deformities can occur during the course of LCPD such as loss of sphericity and increased size of femoral head, decreased centrum-collum-diaphyseal (CCD) angle or femoral neck length, as well as lateral (sub)luxation of the proximal femoral epiphysis from the center of the acetabulum. It is recommended to address these abnormalities surgically [48]. There are two different types of surgical options, containment-restoring procedures or non-containment-restoring procedures. Figure 1 shows a therapy algorithm for LCPD.

### Containment-restoring procedures

Containment-restoring surgery can be performed on the proximal femur by femoral varus osteotomy (FVO) or on the pelvis e.g., as Salter’s innominate osteotomy (SIO) or triple pelvic osteotomy (TPO). After restoration of containment with the epiphysis re-centered in its central position within the acetabular cup, physiologic remodeling of the weight-bearing portion of the proximal femoral epiphysis begins. Resection or correction of the lateral protrusion of the proximal femoral epiphysis is therefore not necessary as it should remodel to physiological states.

In cases of severe decentration and secondary acetabular involvement, the FVO can be combined with a pelvic redirection osteotomy in the sense of “advanced containment”. If the skeletal age is less than 8 years, solely FVO is possible due to the sufficient revalgization potential [50]. FVO has the same biomechanical effect on restoring physiological joint properties compared to a permanent abduction position of the leg. SIO has the effect of a tilt of the upper body and thus of the pelvis to the affected side. In both cases, the lateral portion of the femoral head is centered in the acetabular cup. Better covering of the anterolateral portion of the femoral head can be achieved with the SIO [3]. Though a similar effect can be achieved on the femoral side by adding an extending component to FVO [3]. In comparison TPO has a similar effect as SIO, with better options for redirection. Additionally, it does not increase the pressure in the joint (unlike the SIO). However, regarding surgical treatment an abduction of the affected hip joint of at least 30 ° is required to ensure surgical success. The lack of abduction ability, especially in the case of a FVO, carries the risk of a postoperative adduction contracture in addition to increased decentration of the hip [3]. For children aged > 8 years at onset and Herring classification B and B/C, surgical therapy with FVO or SIO shows significantly better results than non-surgical treatment [48]. Early stages of the disease (condensation or fragmentation stage) have shown to be more advantageous for surgery, as there is more remodeling potential [51]. Recently, femoral head reduction osteotomies (FHRO) have been advocated to enhance the sphericity of the femoral head, decrease the size of coxa magna, and thereby improve joint containment [52, 53]. In many cases, they need to be combined with a periacetabular osteotomy (PAO) to address acetabular dysplasia [54, 55]. This integrated approach combines FHRO with PAO, creating a more effective method of treatment for LCPD. This comprehensive surgical strategy aims to restore femoral head sphericity and achieve a better fit within the acetabulum, ultimately enhancing patient mobility and quality of life.

### Femoral varus osteotomy (FVO)

**Fig. 2:**
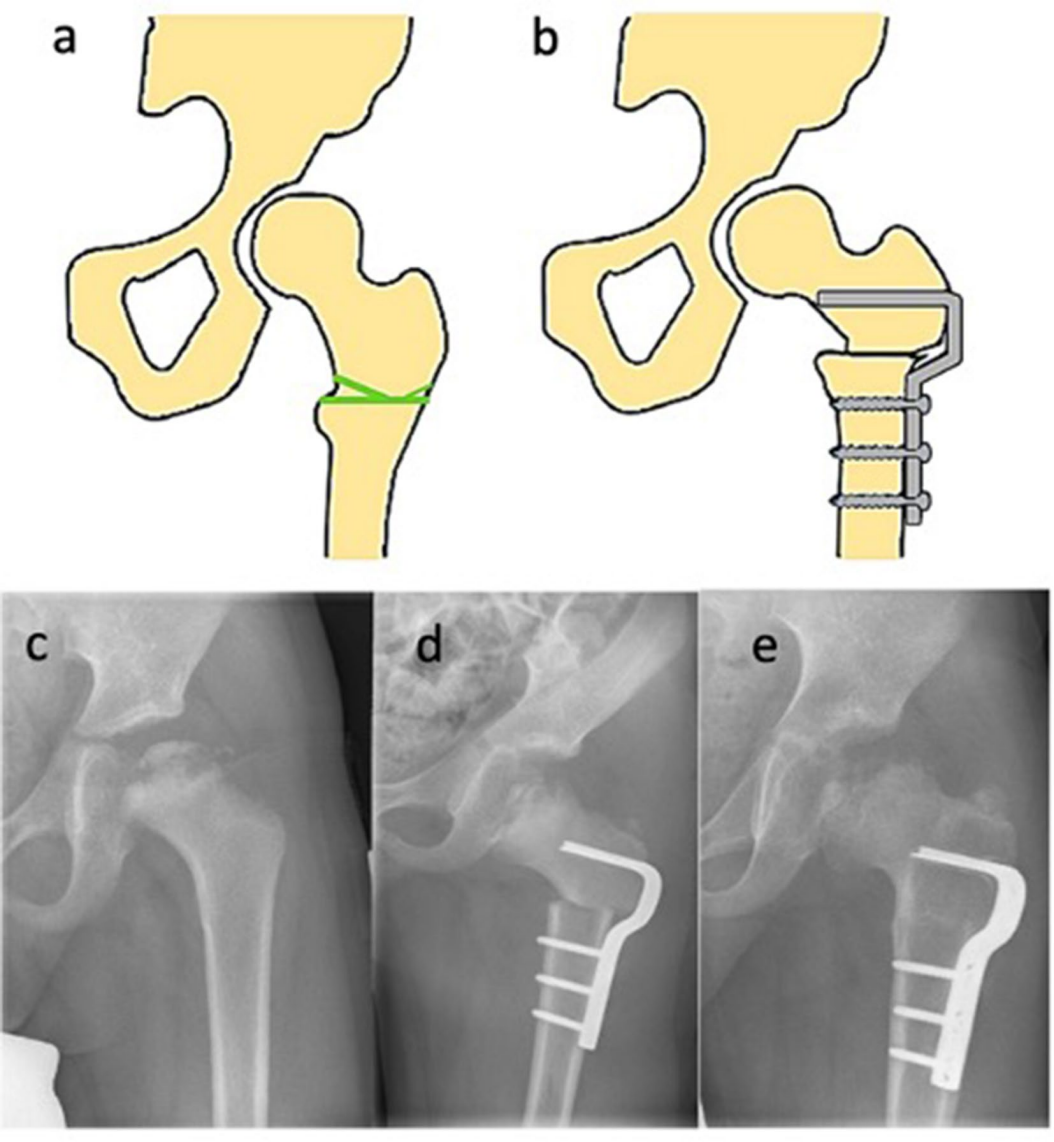
Femoral varus osteotomy (FVO) (3): (**a**) Schematic view of a hip pre-osteotomy. The green line indicates the planned site of the osteotomy, (**b**) Post-osteotomy schematic illustrating the fixation of the femur with a plate, (**c**) Preoperative X-ray showing the hip’s condition prior to FVO, (**d**) Immediate postoperative X-ray demonstrating the repositioning and fixation of the femur following FVO, (**e**) Six-month postoperative X-ray showing the progression of bone healing and alignment following the surgical intervention

FVO is the first and most preferred surgical treatment for LCPD worldwide and shows favorable long-term results [46, 56]. This procedure aims to center the anterolateral portion of the femoral head in the acetabulum and prevent secondary changes. Varization of the proximal femur can be accomplished either by open- or closed wedge technique to improve containment if the femoral head is not sufficiently covered [57]. Cases of extensive femoral anteversion can be addressed with a derotational component to the FVO. Despite the limited internal rotation capacity in children with LCPD, implementing a derotational component to the FVO for addressing extensive femoral anteversion could inadvertently exacerbate the situation, as increased antetorsion is rarely observed in such cases. Furthermore, the stable osteosynthesis of the FVO using blade plates or locking plates offers the possibility of functional follow-up treatment (Fig. 2). Possible unfavorable alteration of this procedures include changes in biomechanical lever of the abductors as well as the offset, varying limb lengths and limping (Table 6) [58]. To reduce the chances of these alterations, it is recommended to combine the procedure with trochanter apophyseodesis or distalization and not to exceed a correction of more than 15° varus [58, 59]. Younger patients do show revalgization at age < 7 years and show the least leg length discrepancy and greatest revalgization with an open wedge technique [60].

**Table 6:** Advantages and disadvantages of surgical procedures for Legg-Calvé-Perthes disease [3, 42, 83, 84]

Surgical procedure	Advantages	Disadvantages
femoral varus osteotomy	• Lower complication rate • Treatment on the affected joint component • Decrease of intraarticular pressure • Tendency to revalgization with skeletal maturity	• Possibility of limb shortening • Relative elevation of the greater trochanter (gluteal insufficiency) • Increasing of femoral offset • Trendelenburg limping • Steep orientation (verticalization) of the physis • Possibility of adduction contracture • Valgus deformity of knee joint
pelvic redirection osteotomies	• Possibility of limb lengthening • No Trendelenburg limping • No change in abductor levers and femoral offset • Improvement of anterolateral covering of the femoral head	• Treatment on the non-affected joint component • Increase of intraarticular pressure (SIO) • Higher complication rates • Iatrogenic femoro-acetabular impingement • Iatrogenic retroversion of the acetabulum TPO)

### Femoral head reduction osteotomy

Ganz et al. [61–63], devised an innovative approach to treat the deformed, non-spherical femoral head resulting from LCPD. They observed that the most severe damage was typically found in the central third of the enlarged femoral head, while the lateral third showed better preservation of articular cartilage. Consequently, they proposed the removal of the affected central part of the femoral head and repositioning the more spherical lateral section to the stable medial segment, forming a more spherical femoral head. This can improve mobility and relieve pain.

Surgical technique [61–63]: The procedure begins with the surgical hip dislocation. An extended retinacular soft tissue flap is developed to maintain the vascularity of the mobile segment of the femoral head. Sagittal osteotomies of the femoral head allow the removal of the necrotic central segment, preserving the femoral head’s blood supply. The mobile fragment, rich in articular cartilage, is then fixed to the stable medial head-neck segment. Careful adjustment is made to ensure joint surface congruity and to avoid joint instability.

In cases of acetabular dysplasia, where the femoral head is often non-spherical, performing only an FHRO may lead to or exacerbate hip instability [55]. To counteract this issue, a concurrent acetabular reorientation, such as TPO or Bernese periacetabular osteotomy (PAO) as described by Ganz, has been suggested to avert further instability [52, 53, 63, 64].

### Pelvic redirection osteotomies

#### Salter’s innominate osteotomy (SIO)

**Fig. 3:**
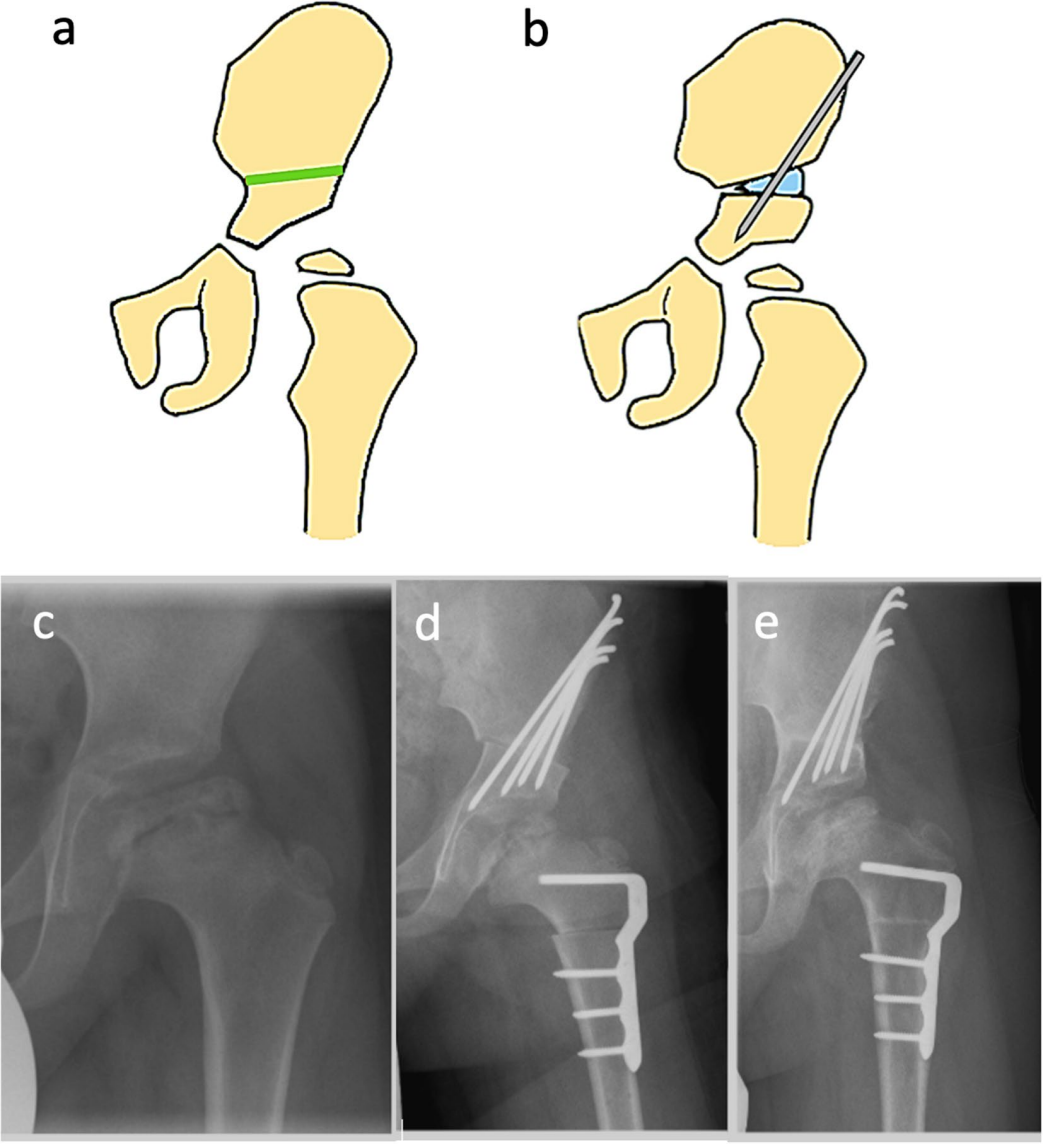
Salter’s innominate osteotomy (SIO) (3) (**a**) Schematic view of a hip pre-osteotomy. The green line indicates the planned site of the osteotomy, (**b**) Post-osteotomy schematic illustrating the fixation of the pelvis with a wire and bone graft, (**c**) Preoperative X-ray showing the hip’s condition prior to SIO, (**d**) Immediate postoperative X-ray demonstrating the repositioning and fixation of the pelvis and femur following SIO and FVO, (**e**) Six-month postoperative X-ray showing the progression of bone healing and alignment following the surgical intervention

The SIO was actually introduced for the treatment of dysplasia of the hip and represents the first pelvic redirection osteotomy used for LCPD [65]. To perform pelvic redirection osteotomies a good hip range of motion, a spherical femoral head, and good joint congruency in abduction are required. During SIO, the pelvis is transected transversely above the spina iliaca anterior inferior to the foramen ischiadicum. The acetabulum is redirected ventrally and laterally and a triangular bone wedge is used to secure the achieved position flattening an excessively steep acetabulum and improving the ventrolateral covering of the femoral head (Fig. 3**)**. The pivot point for the displacement is the symphysis. The advantages of pelvic osteotomy over FVO are the avoidance of leg length shortening and thus the alteration of the lever of the abductors resulting in no limping due to gluteal insufficiency (Table 6). SIO alters the biomechanics of the hip joint through acetabular rotation, which improves femoral head coverage but also influences joint pressure and muscle mechanics [66, 67]. Furthermore, SIO can lead to leg lengthening, as there is distalization of the acetabulum. A biomechanical study by Pfeifer et al. [66] showed that postoperatively, the center of the femoral head shifts medially by approximately 15–16 mm and caudally by around 16 mm. This displacement increases the contact area between the acetabulum and femoral head, thereby reducing localized stress but leading to changes in muscle function. The length of the gluteus medius muscle increases by approximately 8 mm, while the gluteus maximus extends by 5 mm, resulting in reduced force generation. Joint reaction forces also decrease significantly, from a preoperative 270% body weight to 120% postoperatively, highlighting an overall reduction in mechanical loading. The procedure itself may increase the initial intra-articular pressure. This effect is mainly caused by the shortened tendon of the psoas muscle and can be reduced by additional aponeurotic lengthening of the psoas tendon. However, SIO has been associated with mild acetabular retroversion, particularly at the roof level, which may predispose patients to femoro-acetabular impingement (FAI) [68]. These biomechanical alterations must be considered when selecting surgical techniques, particularly in severe LCPD cases where additional procedures, such as FVO, may be required to optimize containment while maintaining favorable load distribution and muscle function [69].

### Triple pelvic osteotomy (TPO)

**Fig. 4:**
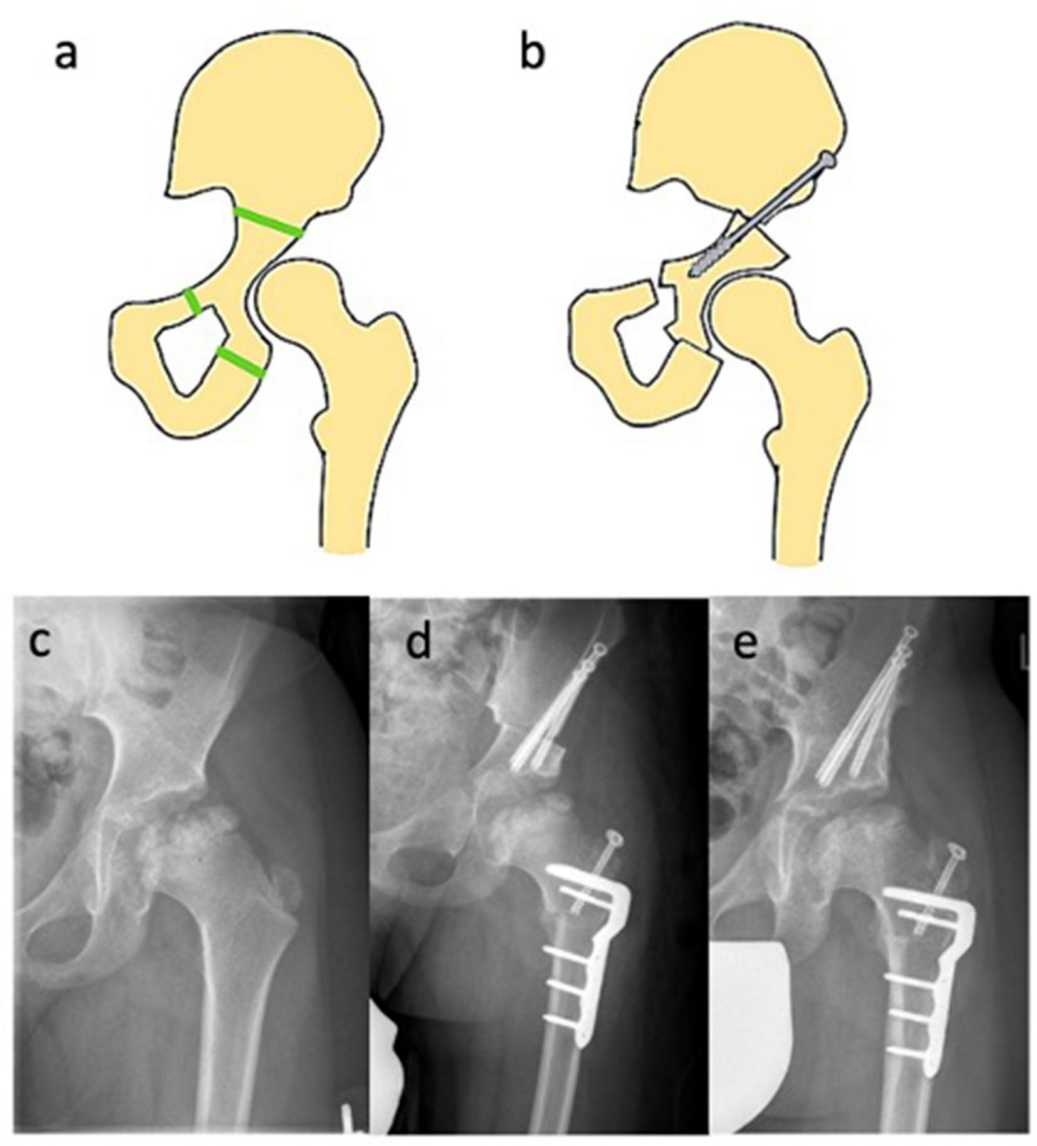
Triple pelvic osteotomy (TPO) (3) (**a**) Schematic view of a hip pre-osteotomy. The green lines indicate the planned site of the osteotomies, (**b**) Post-osteotomy schematic illustrating the fixation of the pelvis with a screw and bone graft, (**c**) Preoperative X-ray showing the hip’s condition prior to TPO, (**d**) Immediate postoperative X-ray demonstrating the repositioning and fixation of the pelvis and femur following TPO, FVO and trochanteric apophyseodesis, (**e**) Six-month postoperative X-ray showing the progression of bone healing and alignment following the surgical intervention

In patients above the age of 8, the acetabulum shows a reduced elasticity. Therefore, TPO aims to completely release the acetabulum from its fixation by three separate osteotomies with subsequent reorientation and refixation [3, 69]. TPO can enhance the containment of the weight-bearing and mechanically important anterolateral region of the hip joint, but comes at the cost of potentially compromising the less important medial portions from a biomechanical perspective. Whilst SIO is only able to improve the containment of lateral parts of the hip joint, TPO allows a wide redirection of the acetabulum, usually antero-laterally, but also dorso-laterally according to the individual condition (Fig. 4**)**. Therefore, the procedure yields a certain risk of overcorrection [70]. When performed in the reconstitution stage TPO alone promises good results in contrast to FVO or SIO [71]. Wenger et al. [69] showed good results in their retrospective study and consider TPO as the treatment of choice in older children. Acetabular osteotomies of the three pelvic bones (pubis, ischium and ilium) are usually performed through three separate surgical approaches. The achieved mobility of the acetabulum necessitates the improvement of the containment of the lateralized femoral head. It is particularly suitable for cases with a severe head involvement, a plump and shortened femoral neck with remaining remodeling potential [71]. The acetabulum and the femoral head should be moderately spherical and congruent. If this is not the case, the femoral head and acetabulum must be corrected simultaneously by the same amount to maintain aspheric congruence. TPO is a challenging surgical procedure and thus carries greater surgical risks and complication rates [72]. TPO is performed using either the Steel’s [73] or Tönnis’ [74, 75] technique. The advantage of Tönnis’ technique is that the osteotomy of the ischial bone is closer to the acetabulum due to the dorsal approach. Overall, this procedure shows a high correction potential with good results, which is also feasible in children with an open Y-joint. TPO shows synergistic effects when combined with FVO in the sense of “advanced containment“ [42].

The Bernese-type triple pelvic osteotomy (BTPO) is an advanced redirectional acetabular osteotomy that merges Ganz periacetabular osteotomy [76] and TPO [74] allowing for extensive acetabular correction in complex cases and achieving superior biomechanical stability [77]. As with other redirectional pelvic osteotomies, BTPO preserves the triradiate cartilage, making it suitable for skeletally immature patients [78]. The BTPO utilizes a single-incision technique instead of the traditional TPO’s three, streamlining the procedure and potentially minimizing surgical complications and recovery time [70, 78]. The incision is made along the anterior third of the iliac crest and the anterior border of the tensor fasciae latae using a modified Smith-Peterson approach [78].

### Lateral shelf procedure

In the 1990s, the slotted acetabular augmentation or lateral shelf procedure became popular in the Anglo-American world as a surgical therapy option for LCPD, in which the acetabulum is augmented according to the deformity of the femoral head [79]. Compared to “real” pelvic osteotomies, the lateral shelf procedure has no advantages. Besides that a major disadvantage is that the acetabular augmentation carries the risk of damaging the acetabular growth plate and that the intraarticular part of the augmentation is not covered with hyaline cartilage [42, 72].

### Periacetabular osteotomy

Periacetabular osteotomy (PAO) is a surgical procedure employed to treat a range of hip conditions, LCPD. The Bernese PAO [80, 81] procedure is a complex yet effective surgical approach to treat hip conditions through a modified Smith-Petersen approach, forgoing exposure of the outer side of the ilium. The five steps of the PAO procedure include the incomplete osteotomy of the ischium, the complete osteotomy of the pubis, and supra-acetabular and infra-acetabular osteotomies. The acetabular fragment is then fully mobilized and reoriented to the desired position. Pelvis X-ray serves as a check for the correction. It’s important to note that the successful execution of PAO requires an exact understanding of pelvic anatomy and careful preoperative planning. In recent years, advanced techniques such as computer-assisted intraoperative navigation have been introduced to increase the precision of the procedure [82].

### Arthrodiastasis

Arthrodiastasis represents an approach in the management of LCPD, focusing on reducing hip joint stress to preserve the structural integrity of the femoral head. A recent systematic review reports significant improvements in hip mobility, with hip flexion increasing from 55.32° to 90°, abduction from 12.28° to 35.28°, internal rotation from 8.69° to 24.93°, and external rotation from 21.73° to 33.71° postoperatively [85]. Most patients achieved Stulberg stages two and three, indicating maintained or improved joint congruency. However, the procedure is associated with manageable complications like pin tract infections. While the available data are limited, making this technique a secondary consideration, it is deemed significant enough to warrant mention for its potential value in the broader management strategy of LCPD.

### Complications and sequelae of LCPD

#### Non-containment-restoring procedures

In cases where hip abduction is significantly limited due to adductor muscle shortening, rather than a true hinge abduction, an adductor tenotomy or release may improve range of motion and is often performed adjunctively with other procedures [48]. This procedure is usually used as an adjunctive measure to other surgical procedures. Due to the involvement of the physis of the proximal femur and the associated relative overgrowth of the trochanter apophysis or as a result of a FVO, trochanter elevation is frequently observed, which is unfavorable for the biomechanical condition of the hip joint. This can be prevented by trochanter apophyseodesis. Functionally, the success of this measure is reflected in a less frequently occurring Trendelenburg sign. The measure should be performed before the age of 9 years, and the indication for this should be generous [42, 86].

### Hinge abduction

**Fig. 5:**
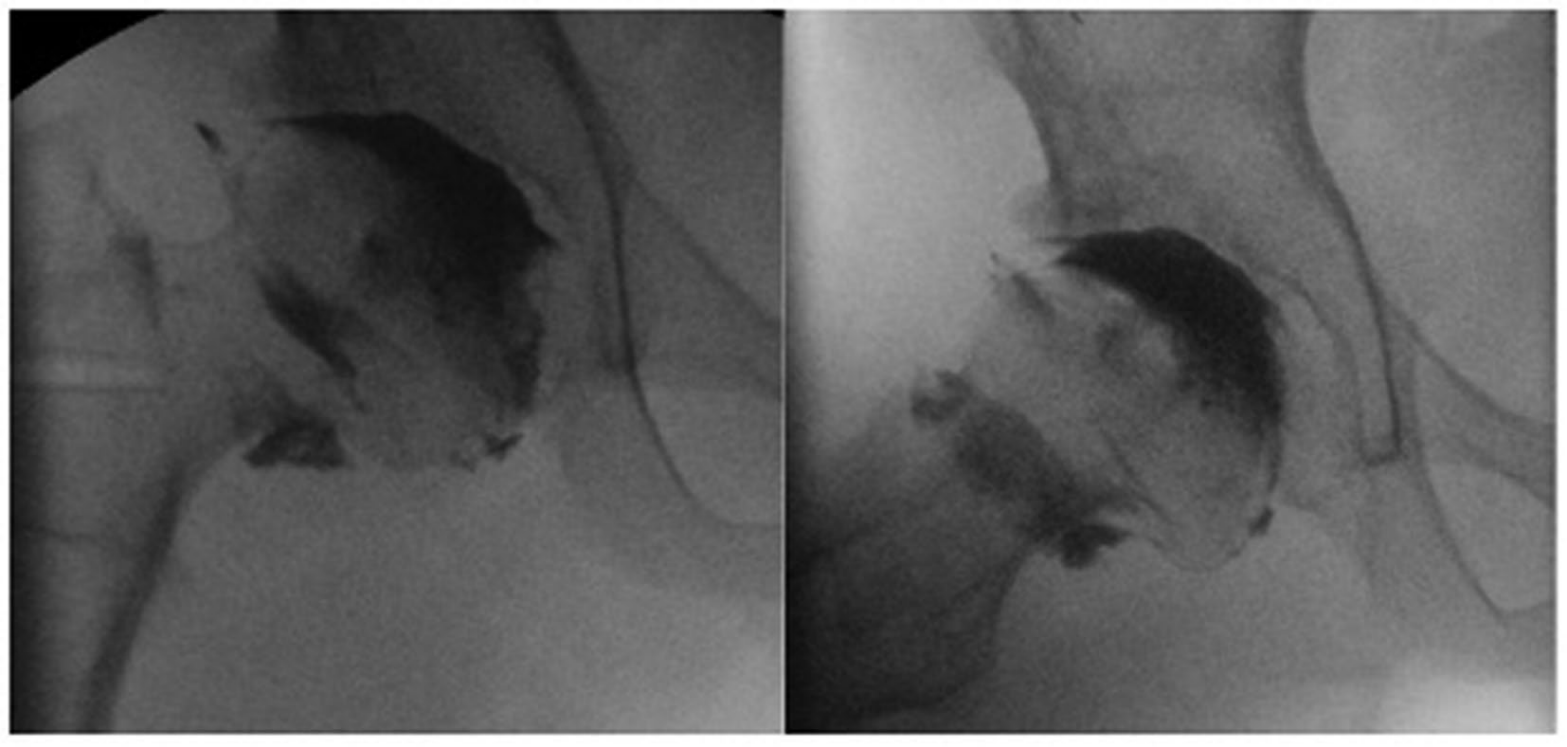
Arthrography: The affected hip joint is visualized in more detail by intra-articular application of contrast medium. These functional images provide a reliable method, in addition to x-ray in abduction, for detecting hinge abduction prior to planned surgery. Furthermore, arthrography provides reliable assessment of the containment and congruence of the head and cup

If the lateral pillar is affected to a higher degree, there may be a reduction in lateral support and increasing lateralization and subluxation. In addition, stress on the epiphysis leads to further deformity. The epiphysis cannot enter the acetabulum during a hinge abduction. The head is levered out by the superolateral parts of the femur hitting the acetabular rim during abduction. It can be well visualized radiologically by medial pooling and displacement of the labrum acetabulare in arthrography [87] (Fig. 5). In the most severe cases, displacement of the center of rotation results in a hinge joint with a typical mushroom shape of the femoral head. In this case, the femoral head is no longer suitable for containment-restoring procedures such as FVO, SIO or TPO. Today, the therapy of choice is a femur valgus extension osteotomy (FVEO) [88–90]. Further salvage procedures can be performed to restore sphericity of the femoral head and to improve the femoro-acetabular containment with resection of the lateral protruding bump or femoral head reduction osteotomy (FHRO) suggested by Ganz et al. [53, 91, 92]. Overall, a hinge abduction is associated with worse outcome [93].

### Coxa vara and trochanter elevation

**Fig. 6:**
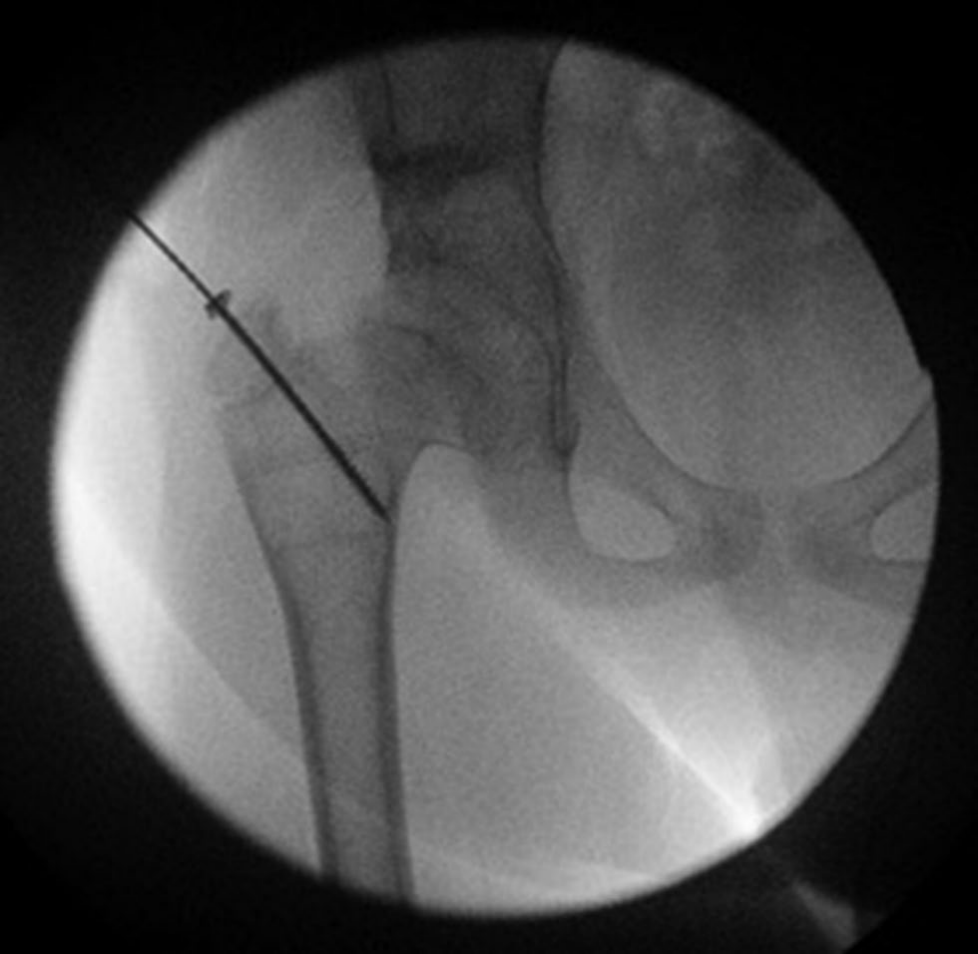
Trochanter apophyseodesis: It is important to ensure that the screw is in a bicortical position

At the endpoint of LCPD, coxa vara with trochanter elevation is a common problem. Functionally, this leads to painful restriction of hip motion and reduced leverage of the hip abductors resulting in gluteal insufficiency (Trendelenburg limp). In order to correct this deformity adequately and to restore the physiological anatomy with correction of the biomechanical leverage, Morscher’s femoral neck lengthening osteotomy (FLO) can be performed [94, 95]. If the CCD angle is maintained, trochanter distalization alone can also be successful [95, 96]. In this case, the femoral neck is not lengthened in a true sense as suggested by Morscher’s procedure. Nevertheless, a relative lengthening of the femoral neck is achieved by trochanter distalization [95, 97]. If the growth plate is still open, the same result can be achieved by trochanter apophyseodesis (Fig. 6) [96]. That should be considered before the age of 9 years, when there is sufficient growth potential that can help optimize hip biomechanics.

### Femoral valgus extension osteotomy (FVEO)

**Fig. 7:**
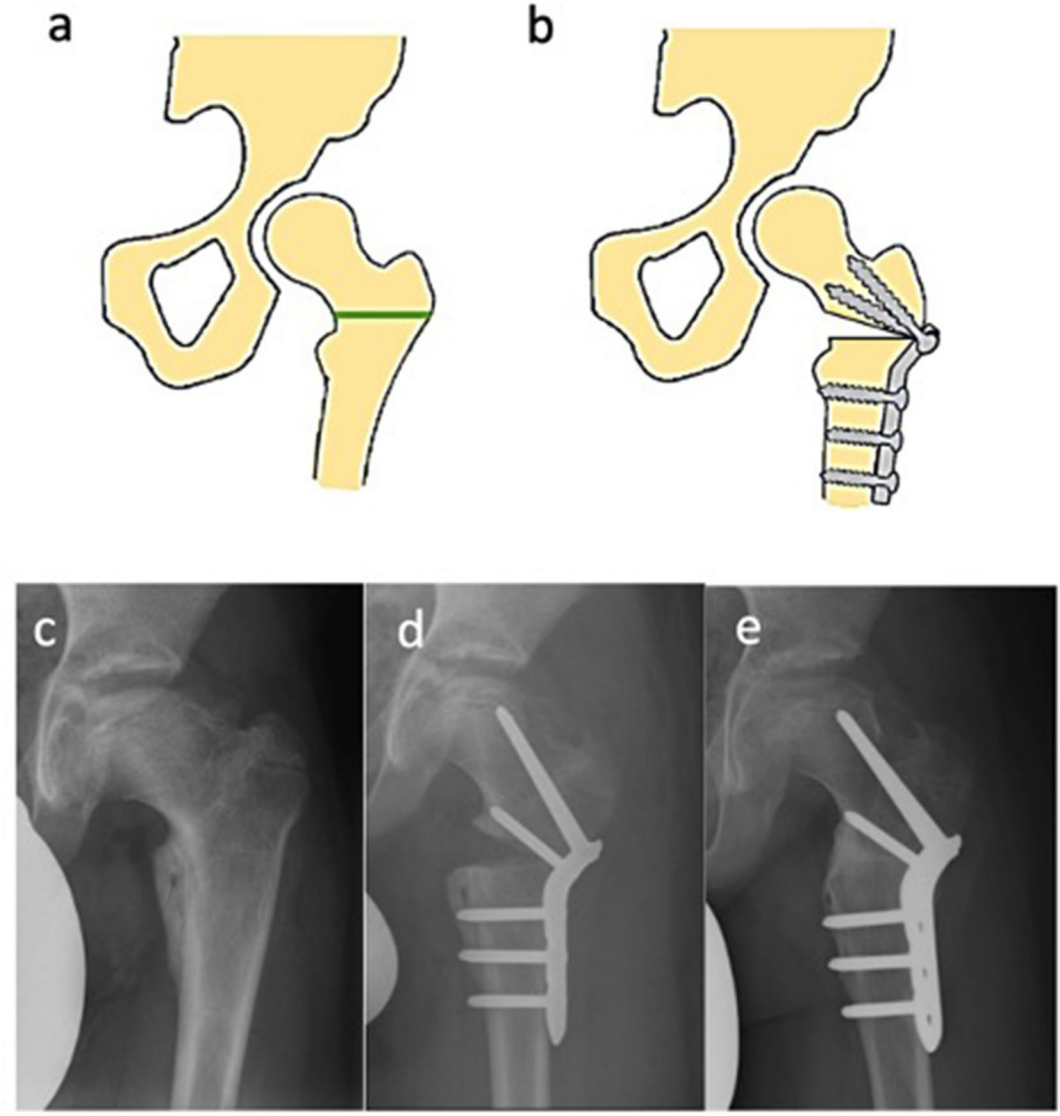
Femoral valgus extension osteotomy (FVEO) (3) (**a**) Schematic view of a hip pre-osteotomy. The green line indicates the planned site of the osteotomy, (**b**) Post-osteotomy schematic illustrating the fixation of the femur with a plate, (**c**) Preoperative X-ray showing the hip’s condition prior to FVEO, (**d**) Immediate postoperative X-ray demonstrating the repositioning and fixation of the following FVEO, (**e**) Six-month postoperative X-ray showing the progression of bone healing and alignment following the surgical intervention

FVEO is a procedure to address coxa vara or hinge abduction phenomena in LCPD, (Fig. 7**)**. The aim of this procedure is to integrate the medial, better-preserved part of the femoral head into the loading zone, reduce adduction contracture, distalize the greater trochanter, which can functionally lengthen the femoral neck and improve biomechanical conditions of the abductor muscles [98–100].

### Morscher’s femoral neck lengthening osteotomy (FLO)

**Fig. 8:**
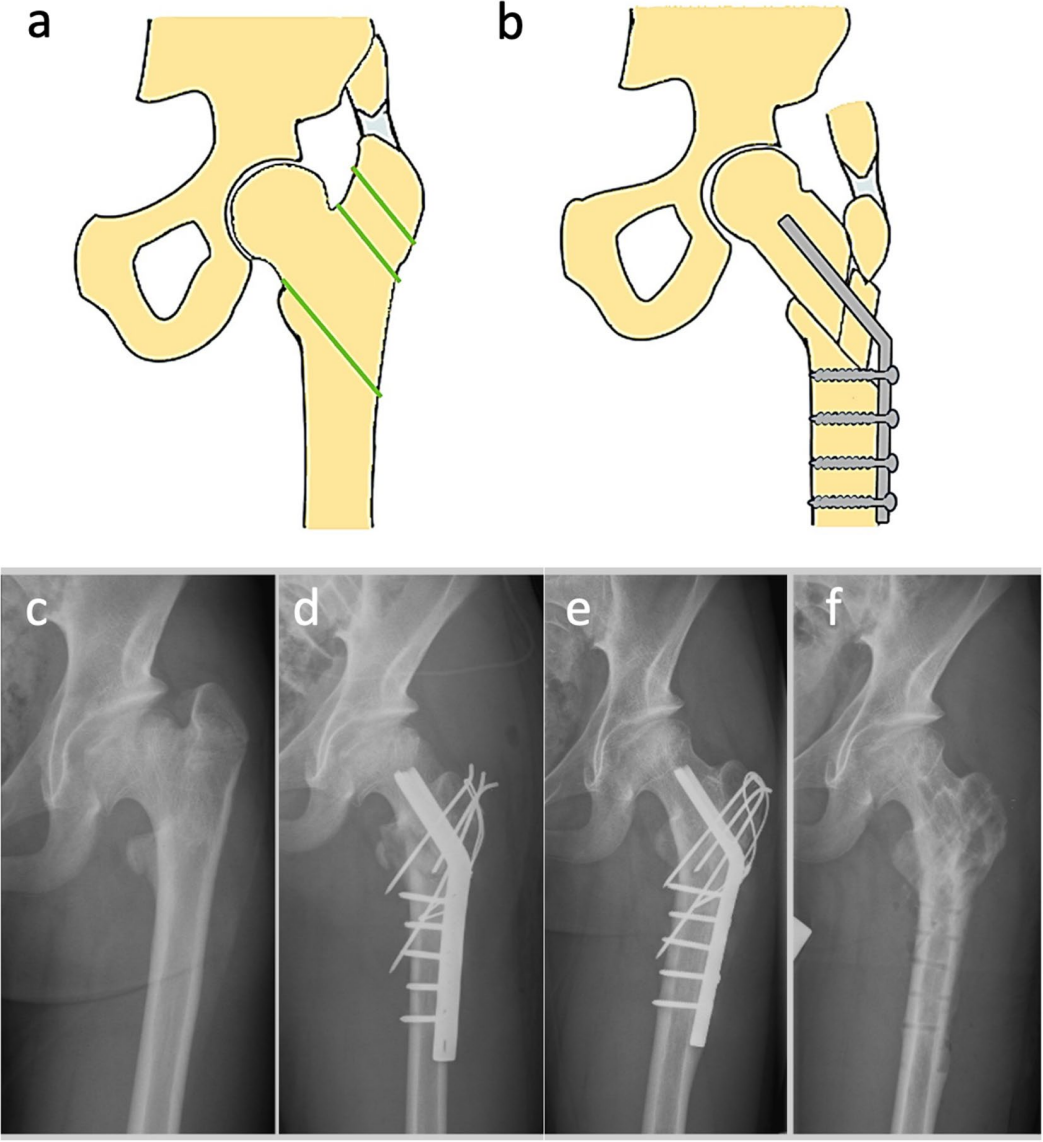
Morscher’s femoral neck lengthening osteotomy (FLO) (3) (**a**) Schematic view of a hip pre-osteotomy. The green lines indicate the planned site of the osteotomy, (**b**) Post-osteotomy schematic illustrating the fixation of the femur with a plate, (**c**) Preoperative X-ray showing the hip’s condition prior to FLO, (**d**) Immediate postoperative X-ray demonstrating the repositioning and fixation of the femur following FLO, (**e**) Six-month postoperative X-ray showing the progression of bone healing and alignment following the surgical intervention, (**f**) Postoperative X-ray after implant removal

A typical consequence of LCP is shortening of the femoral neck with simultaneous overlength of the greater trochanter, since the trochanteric apophysis is usually not affected by the necrosis, leading to a coxa brevis [1]. The femoral neck lengthening osteotomy according to Morscher [95] can be performed to address coxa brevis (Fig. 8**)**. It aims to restore a normal and physiological anatomy and length of the femoral neck, to compensate the leg length difference, and to improve the lever arm of the hip abductors leading to a normalization of gait patterns [94, 95].

### Femoro-acetabular impingement (FAI)

Another late consequence or sequalae of LCPD may be anterolateral FAI, which is caused by coxa vara et magna or due to an aspheric head configuration [101]. Surgical hip dislocation with trimming of the head or head-neck junction is a possible treatment option [91, 92, 102]. This technique allows correction of the actual pathology. Alternatively, minimally invasive arthroscopic techniques for head trimming and labral surgery are available with significant improvement in hip function and pain reduction [101, 103]. However, in case of impingement due to coxa magna et plana caused by LCPD, the bump is covered by cartilage and bump resection is thus damaging to a certain extent.

Minimally invasive hip arthroscopy has emerged as a viable treatment option for managing FAI and associated intra-articular pathology in patients with LCPD. Studies suggest that hip arthroscopy can effectively address labral tears, chondral lesions, and osteochondral fragments, offering significant symptom relief and functional improvement [101]. Compared to open procedures, arthroscopy preserves joint integrity, reduces recovery time, and minimizes the risk of avascular necrosis [101]. However, its indications remain limited to cases with mild to moderate deformity, as severe anatomical alterations may necessitate corrective osteotomies or THA.

Other non-containment-restoring procedures are available for LCPD, but they are of little or no importance in clinical practice today and vastly abandoned: Chiari osteotomy [104], and arthrodiastasis using external fixator [105].

### Sequelae of Legg-Calvé-Perthes disease

As patients with LCPD experience a 5% incidence of THA 20–30 years after surgical treatment (approximately 2.4% after pelvic redirection surgeries and 8.6% after proximal femoral osteotomies), and more than 15% after 40 years of follow-up, those with a history of surgical interventions for LCPD present unique challenges when undergoing THA due to significant anatomical alterations and biomechanical changes [106]. These include coxa magna, coxa brevis, FAI, metaphyseal-diaphyseal mismatch, and a high-riding trochanter, all of which can complicate implant positioning and stability [107, 108]. Additionally, previous osteotomies, such as FVO and pelvic redirection osteotomies, introduce further complexities due to altered bone stock, residual deformities, and scar tissue, which may limit surgical exposure and increase the risk of intraoperative fractures [108]. One of the primary concerns in THA for LCPD patients is the sclerotic and misshapen proximal femur, which makes reaming for a standard femoral component difficult. This can lead to complications such as femoral perforation, inadequate fixation, and increased risk of periprosthetic fractures [107, 108]. Modular and custom femoral implants have been proposed as effective solutions, allowing for intraoperative adjustments in neck length, version, and offset, while also addressing metaphyseal-diaphyseal mismatches [108]. A systematic review by Hanna et al. reported the use of different stem designs in THAs, of which 90% were cementless, 9% hybrid, and 1% cemented, including 76% standard stems, 18% modular stems, and 6% custom-made stems [107]. Previous studies suggest that modular femoral components reduce the risk of intraoperative fractures compared to standard broached stems, though concerns about taper fretting and corrosion remain. Cementless monoblock stems have also shown favorable outcomes but carry a higher risk of intraoperative fractures if not carefully selected for individual patients [109].

Another critical issue is leg length discrepancy (LLD), which is frequently encountered due to the shortening effects of LCPD and previous osteotomies (e.g., FVO). Significant limb lengthening during THA increases the risk of sciatic nerve palsy, with studies reporting neurological complications in up to 3–6% of cases—higher than in primary THA for osteoarthritis [107, 108]. Shortening osteotomies may be necessary in cases with excessive preoperative LLD to reduce this risk [108]. Additionally, the altered morphology of the acetabulum following pelvic osteotomies, such as SIO or TPO, can result in acetabular retroversion, compromised coverage, and impingement, necessitating careful acetabular component placement.

Despite these challenges, THA remains an effective treatment for end-stage hip degeneration in LCPD patients, with studies demonstrating significant improvements in function and patient satisfaction [107]. Despite comparable long-term survivorship to THA patients without LCPD, the revision rate is slightly higher compared to primary THA for osteoarthritis, with aseptic loosening and periprosthetic fractures being the most common failure modes [107, 108]. Proper preoperative templating, the use of navigation or robotic assistance, and meticulous intraoperative techniques can be helpful to optimizing outcomes. Given the complexity of these cases, patients should be counseled extensively on potential risks, including the possibility of requiring a staged or revision procedure to address residual deformities or complications.

Table 7 provides a comprehensive comparative overview of different surgical approaches for LCPD.

**Table 7:** Comparative analysis of surgical procedures for Legg-Calvé-Perthes disease [41, 110, 111]

Surgical Procedure	Indications	Expected Outcomes	Complications and Risks	Comparison with Other Procedures
Femoral Varus Osteotomy (FVO)	• Patients < 8 years • Herring B, B/C • good range of motion • Early surgical option • widely used • initial fragmentation stage	• Improves containment • reduces femoral head deformation • corrects flexion and rotational deformities	• Limb length discrepancy (shortening) • Trendelenburg gait • excessive varus positioning (exceeding 15° of varus)	• good outcomes for B, B/C • Less invasive than TPO/SIO • may lead to gait abnormalities and leg length discrepancy
Salter’s Innominate Osteotomy (SIO)	• Patients < 8 years • Herring B, B/C • initial fragmentation stage • good congruence • Strong clinical evidence • allows acetabular redirection	• Improves femoral head coverage and congruency (remodeling of femoral head during growth) • Reduces biomechanical stress over hip joint	• Overcorrection risk • acetabular impingement • delayed union • increased intra-articular pressure • risk of acetabular retroversion	• good outcomes for B, B/C • Less invasive than TPO • may have higher risk of impingement • similar outcomes regarding femoral sphericity compared to FVO • increased femoral head coverage compared to FVO
Triple Pelvic Osteotomy (TPO)	• Severe containment loss • lateral pillar C • older patients • Prevents progression to osteoarthritis • better joint preservation	• Enhances femoral head coverage • improves joint congruency	• Technically demanding • risk of overcorrection • post-op stiffness • risk of pincer impingement	• More effective than SIO/FVO in severe cases • More acetabular rotation than SIO • Less leg length discrepancy than FVO • Higher rate of complications
Periacetabular Osteotomy (PAO)	• Older patients (> 10 years) • acetabular dysplasia • Preserves native joint	• Improves hip joint stability • delays need for THA	• Complex surgery • prolonged recovery • risk of nerve injury	• Preferred over TPO in adolescents and young adults with dysplasia
Combined Femoral and Pelvic Osteotomy	• Severe cases • Patients > 8 years • lateral pillar B/C • poor containment • Maximizes coverage, preserves joint • improves femoral head congruency • Synergistic effect enhances containment more than isolated procedures	• Improved joint congruence • lower osteoarthritis risk • better long-term function	• Longer recovery • risk of leg-length discrepancy	• More effective in severe cases or in children with higher age • requires prolonged rehabilitation • reduction of increased intra-articular pressure (SIO) and compensation of leg shortening (FVO)
Femoral Head Reduction Osteotomy (FHRO)	• Advanced femoral head deformity • hinge abduction • delays THA	• Improves hip function • prevents femoro-acetabular impingement	• Risk of avascular necrosis of femoral head • Progression • long rehabilitation period	• More effective than SIO/FVO alone in treating hinge abduction
Trochanteric Apophyseodesis	• Prevents excessive femoral overgrowth • used in combination with other procedures • Simple procedure • minimal invasiveness	• Reduces risk of Trendelenburg gait • prevents trochanteric overgrowth	• Residual abductor weakness • delayed bone healing	• Often used adjunctively with FVO to prevent overgrowth • Should be performed before the age of 9 years
Arthrodiastasis (Joint Distraction)	• Severe cases with joint collapse • poor containment • Alternative to osteotomy • preserves joint function • alternative treatment method	• Reduces intra-articular pressure • delays THA	• Infection risk (pins) • prolonged fixation • limited availability	• Useful for joint salvage in non-containable cases
Chiari Osteotomy	• Late-stage disease • poor containment • severe subluxation • Useful in older children • preserves hip function • popular salvage procedure	• Provides femoral head coverage • improves joint stability • reduces joint loading by medialization	• High surgical complexity • risk of hip stiffness	• Alternative to THA in skeletally mature patients
Total Hip Arthroplasty (THA)	• Skeletally mature patients with severe deformity • Definitive treatment • high success rate in adults	• Restores function • relieves pain	• High revision risk in young patients • implant longevity concerns	• Often complicated by prior osteotomies, which may alter acetabular and femoral anatomy

## Conclusion

Since Perthes disease can vary greatly in severity, there is no standardized therapy algorithm. Individualized therapy must be adjusted to each patient, taking radiographic classification, risk signs, skeletal age, and mobility of the affected hip into account. The initial aim of the therapy should always be the preservation of hip range of motion. Depending on the further course of the disease and the presence of poor prognostic indicators, surgical interventions to restore the sphericity of the femoral head and improve containment of the joint might be indicated. Young patients often have good outcomes with nonoperative treatment; however, in the presence of head-at-risk signs or progressive loss of range of motion, surgical treatment might be indicated. Moderate forms of LCPD show good results when treated with either FVO or SIO. Severe cases are recommended to be treated with HRO or FVO combined with SIO, TPO, or PAO alone to provide good outcomes. LCPD is still a great challenge today with not always satisfactory therapeutic success. The selection of the most appropriate treatment is challenging and depends primarily on the patient’s age at disease onset, radiological risk signs, and the extent of necrosis. Therapy above the age of 8 is more complex, caused by low remodeling potential. Given the excellent results of modern THA, severe anatomy-changing procedures like Morscher’s femoral neck lengthening osteotomy are usually no longer indicated.

## Data Availability

No datasets were generated or analysed during the current study.
